# Establishment and Verification of a Predictive Model for Node Pathological Complete Response After Neoadjuvant Chemotherapy for Initial Node Positive Early Breast Cancer

**DOI:** 10.3389/fonc.2021.675070

**Published:** 2021-04-29

**Authors:** Jiujun Zhu, Dechuang Jiao, Min Yan, Xiuchun Chen, Chengzheng Wang, Zhenduo Lu, Lianfang Li, Xianfu Sun, Li Qin, Xuhui Guo, Chongjian Zhang, Jianghua Qiao, Jianbin Li, Zhimin Fan, Haibo Wang, Jianguo Zhang, Yongmei Yin, Peifen Fu, Cuizhi Geng, Feng Jin, Zefei Jiang, Shude Cui, Zhenzhen Liu

**Affiliations:** ^1^ Department of Breast Disease, Henan Breast Cancer Center, Affiliated Cancer Hospital of Zhengzhou University, Henan Cancer Hospital, Zhengzhou, China; ^2^ Department of Breast Oncology, The Fifth Medical Center of Chinese PLA General Hospital, Beijing, China; ^3^ Department of Breast Surgery, The First Hospital of Jilin University, Changchun, China; ^4^ Department of Breast Cancer Center, Affiliated Hospital of Medical College Qingdao University, Qingdao, China; ^5^ Department of Breast Surgery, The Second Affiliated Hospital of Harbin Medical University, Harbin, China; ^6^ Department of Breast Cancer, Jiangsu Province Hospital and Nanjing Medical University First Affiliated Hospital, Nanjing, China; ^7^ Department of Breast Center, First Affiliated Hospital, School of Medicine, Zhejiang University, Hangzhou, China; ^8^ Department of Breast Cancer Center, Hebei Medical University Fourth Affiliated Hospital and Hebei Provincial Tumor Hospital, Shijiazhuang, China; ^9^ Department of Breast Surgery, The First affiliated Hospital of China Medical University, Shenyang, China

**Keywords:** breast neoplasm, lymph node, pathological complete response, predictive model, neoadjuvant chemotherapy (NCT)

## Abstract

**Objective:**

Axillary node status after neoadjuvant chemotherapy (NCT) in early breast cancer patients influences the axillary surgical staging procedure. This study was conducted for the identification of the likelihood of patients being node pathological complete response (pCR) post NCT. We aimed to recognize patients most likely to benefit from sentinel lymph node biopsy (SLNB) following NCT and to reduce the risk of missed detection of positive lymph nodes through the construction and validation of a clinical preoperative scoring prediction model.

**Methods:**

The existing data (from March 2010 to December 2018) of the Chinese Society of Clinical Oncology Breast Cancer Database (CSCO-BC) was used to evaluate the independent related factors of node pCR after NCT by Binary Logistic Regression analysis. A predictive model was established according to the score of considerable factors to identify ypN0. Model performance was confirmed in a cohort of NCT patients treated between January 2019 and December 2019 in Henan Cancer Hospital, and model discrimination was evaluated via assessing the area under the receiver operating characteristic (ROC) curve (AUC).

**Results:**

Multivariate regression analysis showed that the node stage before chemotherapy, the expression level of Ki-67, biologic subtype, and breast pCR were all independent related factors of ypN0 after chemotherapy. According to the transformation and summation of odds ratio (OR) values of each variable, the scoring system model was constructed with a total score of 1–5. The AUC for the ROC curves was 0.715 and 0.770 for the training and the validation set accordingly.

**Conclusions:**

A model was established and verified for predicting ypN0 after chemotherapy in newly diagnosed cN+ patients and the model had good accuracy and efficacy. The underlined effective model can suggest axillary surgical planning, and reduce the risk of missing positive lymph nodes by SLNB after NCT. It has great value for identifying initial cN+ patients who are more appropriate for SLNB post-chemotherapy.

## Introduction

Sentinel lymph node biopsy (SLNB) is considered to be the standard method for the management of axillary nodes in patients with clinical lymph node-negative (cN0) early breast cancer (BC). Neoadjuvant chemotherapy (NCT) is widely used in locally advanced BC, triple-negative (TN), and human epidermal growth factor receptor-2 positive (HER2+) BC (1). NCT elevates the potency that a patient may experience breast-conserving therapy, could significantly downstage the axilla, and permits for an *in-vivo* evaluation of treatment effect. The probability of nodal negativity post NCT affects the choice of axillary staging operation. In patients with cN0 disease before treatment, the feasibility of SLNB after chemotherapy has been confirmed and agreed upon. But in patients who were downstaged from initial lymph node-positive (cN+) disease before treatment to clinically node-negative after chemotherapy, although the safety of SLNB has also been confirmed, while it is still one of the focus of controversy (2). The main reason for the controversy is the concern about patients with negative sentinel lymph nodes but missed metastatic nodes checking post NCT.

The risk of missed detection of metastatic nodes in the population was positively correlated with the false-negative rate (FNR) of SLNB and the load of axillary lymph node metastasis after NCT. Multiple considerable studies have been directed to a modification in the manner surgeons manage the axilla in NCT treated patients of BC. According to the reported trails studies of the American College of Surgeons Oncology Group (ACOSO) Z1071 trial, the SN FNAC trial, and the SENTINA trial, SLNB is considered to be a relatively safe and feasible procedure after NCT. Although the overall FNR was 12.6, 8.4, and 14.2%, respectively (3–5). In the retrospective study, the FNR of SLNB was found to be as high as 5–25% (6, 7). It can be seen that SLNB after NCT still has a higher FNR. On the one hand, FNR could be lowered *via* improving the surgical technique of SLNB, including double tracer with the use of dye combined with a radionuclide, placement of marker clip in positive lymph nodes pre-NCT and its removal during operation, detection of more than two sentinel lymph nodes, and examined the nodes with immunohistochemical method (8). On the other hand, we can use optimizing patient selection. SLNB should be performed in patients associated with a low load of axillary lymph node metastasis or even no node metastasis after chemotherapy. When allowing for SLNB surgery after NCT for a patient associated with cN+ complication at diagnosis, this approach is beneficial for those patients who mostly have a complete nodal response. Ideally, doctors can identify which patients will respond to chemotherapy. They select patients who can most likely achieve node pathological complete response (pCR, ypN0) and suitable to SLNB after NCT. In the same way, the risk of missing positive lymph nodes will be reduced.

Preoperative models that predict the likelihood of the patient achieving a pCR in the axilla after NCT are helpful to guide this decision making. Multiple models have been published predicting axillary pCR after NCT in various cohorts. However, these established models were limited because of single-institution experiences or multicenter small sample size of the studies (9–11). Only three models reported in two studies were based on the materials of the National Cancer Data Base (NCDB) (12, 13). This study plans to develop a clinical preoperative scoring prediction model for the identification of the likelihood of patients being axillary pCR after NCT based on CSCO-BC and to verify the model based on independent data in Henan Cancer Hospital. It is expected to establish and verify a model for predicting ypN0 in newly diagnosed cN+ patients after chemotherapy, to guide axillary surgical planning, and identify initial cN+ patients who are more appropriate for SLNB after chemotherapy, and to achieve the goal of reducing the risk of missing detection of metastatic nodes.

## Materials and Methods

### Study Population

After the approval of the Institutional Review Board, we recognized all those patients with primary BC and obtained preoperative chemotherapy in the CSCO BC database from March 2010 to December 2018 and Henan Cancer Hospital from January 2019 to December 2019. CSCO BC database is an authoritative cancer registry database that contains anonymized BC cases from nine large hospitals representing all regions of China. Variables include patient sex, age at the time of diagnosis, the status of menstruation, location of primary breast tumor, pathological type, estrogen receptor (ER), progesterone receptor (PR), HER2, Ki-67, mode of operation, cTNM stage, axillary pathology, and postoperative breast pathology, etc.

### Inclusion and Exclusion Criteria

**Figure 1:**
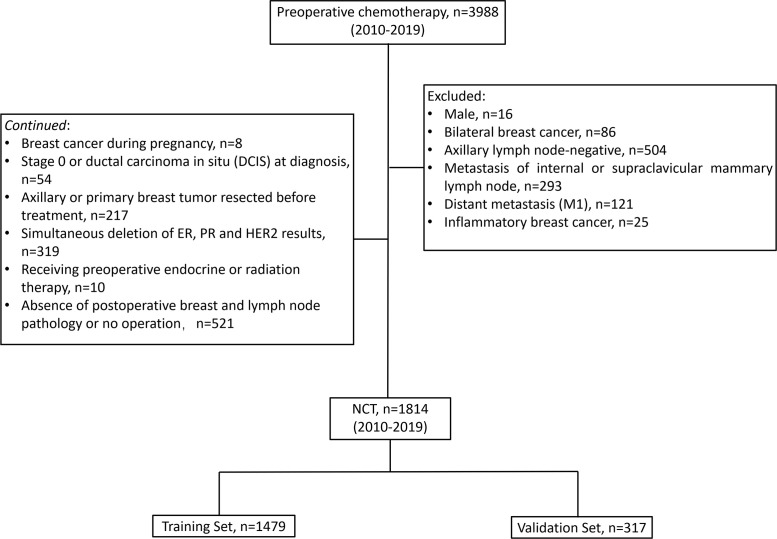
Patient flow diagram. NCT, neoadjuvant chemotherapy; ER, estrogen receptor; PR, progesterone receptor; HER2, human epidermal growth factor receptor-2.

In the existing study, the following conditions were considered as inclusion criteria (1) cTNM stage based on the 7^th^ edition of American Joint Cancer Commission (AJCC) cTNM staging before treatment available; (2) before chemotherapy, invasive BC validated *via* core needle biopsy; (3) axillary lymph nodes positive at diagnosis (cN+); (4) known ER, PR, HER2, Ki-67 status before chemotherapy; (5) received preoperative chemotherapy; (6) received axillary lymph node dissection after chemotherapy, the patient subjected to breast surgery following the local treatment standards; (7) Postoperative pathology of axillary lymph nodes and breast available. Patients with any of the following conditions were not selected for the study: male, bilateral BC, axillary lymph node-negative (cN0), metastasis of internal or supraclavicular mammary lymph node, distant metastasis (M1), inflammatory BC, BC during pregnancy, stage 0 or ductal carcinoma *in situ* (DCIS) at diagnosis, axillary or primary breast tumor resected before treatment, simultaneous deletion of ER, PR, and HER2 results, receiving preoperative endocrine or radiation therapy, absence of postoperative breast and lymph node pathology or no operation. After screening, 1,814 patients were deemed to be eligible and included in the final analysis. The resulted of 1,497 patients from the CSCO BC database being assigned to a ‘‘training set’’ (used in creating our initial model of post-NCT ypN0) and the resulted of 317 patients from Henan Cancer Hospital being assigned to a “validation set” for confirmation of model strength (**Figure 1**).

### Pathology

The immunohistochemistry (IHC) technique was used in order to assess the HER2, Ki-67, ER, and PR status at diagnosis. The ER/PR+: ≥1% of tumorous cells were evaluated with nuclear staining. ER or PR+ were collapsed into one HR+. The determination of HER2+ was carried out based on the ASCO/CAP suggested guidelines. A 3+ score for IHC was considered positive, as was a 2+ IHC score with the results of fluorescence in situ hybridization (FISH) overruled (14). The expression of Ki-67 was categorized as high (>30%) and low (<30%) based on the nuclear positive cells ratio to all tumor cells in 10 high-power visual fields. Based on HR and HER2 status, the patients were divided into four sub-types: HR+HER2−, HR+HER2+, HR−HER2−, and HR−HER2+. The pCR was defined as the absence of tumor cells in the axillary lymph nodes (axillary pCR) or the breast (breast pCR) after NCT (1).

### Statistical Analyses

Statistical analyses were conducted with SPSS, version 23. Categorical variables were compared *via* univariate logistic regression in the training set. Factors that were statistically significant at the 0.1 level were included in the multivariate analysis. Binary logistic regression analysis was employed for multivariate analysis in the training set. ORs and 95% CIs were measured. Odds ratio (OR) >1 indicated an elevated likelihood of pN0. The odds ratios of significant independent predictors were employed for translating into points for the model. The receiver operating characteristic (ROC) curve was plotted, and the predictive accuracy was evaluated *via* measuring the area under the ROC curve (AUC). A 95% CI was measured for all AUC, and was compared with an AUC of 0.5 by Z test. The model is validated in the validation set.

## Results

### Patient Characteristics

Baseline clinical, as well as pathologic properties of the study participants in the training and validation sets, have been revealed in **Table 1**. A total of 1,814 female BC patients were registered in the current study, having 1,497 and 317 in the training and the verification set, respectively. In the training set, the patient’s age was ranged from 19 to 77 years, with an average of 48 years. The age of the patients in the verification set ranged from 22 to 75 years, with an average of 49 years. The clinical T categories were cT1/T2 and cT3/T4 in 1225 (68.2%) and 570 patients (31.7%). Most of the patients were cN1 at presentation [906 (50.3%) cN1 and 609 (33.8%) cN2], while 286 (15.9%) were cN3. In the majority of the patients *i.e.*, 72.1%, expression of Ki-67 was elevated in tumors. Biologic subtype was ER+/HER2- in 42.7% of patients, ER+/HER2+ in 25.4%, ER−/HER2+ in 15.0%, and ER−/HER2− in 16.9%. At surgery, 472 (26%) patients got breast pCR after NCT, having 384 (25.7%) and 88 (27.8%) in the training set and verification set, accordingly. Post chemotherapeutic treatment, 724 (39.9%) patients had no axillary metastasis with 594 (39.7%) and 130 (41%) in training and verification set, accordingly.

**Table 1:** The features of patients for the training as well as a validation set, n (%).

Characteristics		Training Set			Validation set		
		ypN0	ypN+	Total	ypN0	ypN+	Total
Age at diagnosis, years							
	≤40	110 (43.1)	145 (56.9)	255 (100)	25 (39.7)	38 (60.3)	63 (100)
	>40	453 (38.5)	724 (61.5)	1,177 (100)	105 (41.3)	149 (58.7)	254 (100)
Menopausal							
	Premenopausal	384 (40.6)	561 (59.4)	945 (100)	82 (41.6)	115 (58.4)	197 (100)
	Postmenopausal	194 (36.9)	332 (63.1)	526 (100)	48 (40)	72 (60)	120 (100)
Quadrant							
	Upper outer	266 (41.6)	373 (58.4)	639 (100)	68 (39.1)	106 (60.9)	174 (100)
	Lower outer	50 (35.7)	90 (64.3)	140 (100)	21 (50)	21 (50)	42 (100)
	Lower inner	28 (37.3)	47 (62.7)	75 (100)	10 (43.5)	13 (56.5)	23 (100)
	Upper inner	112 (41.6)	157 (58.4)	269(100)	25 (41.7)	35 (58.3)	60 (100)
	Central	38 (33.6)	75 (66.4)	113 (100)	6 (33.3)	12 (66.7)	18 (100)
Clinical T stage							
	T1	44 (43.1)	58 (56.9)	102 (100)	17 (51.5)	16 (48.5)	33 (100)
	T2	349 (40.3)	517 (59.7)	866 (100)	93 (41.5)	131 (58.5)	224 (100)
	T3	138 (38.5)	220 (61.5)	358 (100)	13 (33.3)	26 (66.7)	39 (100)
	T4	55 (35.9)	98 (64.1)	153 (100)	7 (35)	13 (65)	20 (100)
Clinical N stage							
	N1	330 (44.3)	415(55.7)	745 (100)	71 (44.1)	90 (55.9)	161 (100)
	N2	176 (33.6)	348 (66.4)	524 (100)	31 (36.5)	54 (63.5)	85 (100)
	N3	83 (38.4)	133 (61.6)	216 (100)	28 (40)	42 (60)	70 (100)
Ki-67							
	Low	103 (27.6)	270 (72.4)	373 (100)	25 (26.9)	68 (73.1)	93 (100)
	High	435 (44.3)	548 (55.7)	983 (100)	105 (46.9)	119 (53.1)	224 (100)
Subtype							
	HR+HER2−	157 (24.8)	477 (75.2)	634 (100)	31 (23.1)	103 (76.9)	134 (100)
	HR+HER2+	164 (47.1)	184 (52.9)	348 (100)	53 (49.1)	55 (50.9)	108 (100)
	HR−HER2+	135 (56.3)	105 (43.8)	240 (100)	22 (73.3)	8 (26.7)	30 (100)
	HR−HER2−	132 (51)	127 (49)	259 (100)	24 (53.3)	21 (46.7)	45 (100)
Breast pCR							
	No	330 (29.6)	783 (70.4)	1,113 (100)	64 (27.9)	165 (72.1)	229 (100)
	Yes	264 (68.8)	120 (31.3)	384 (100)	66 (75)	22 (25)	88 (100)
Total		594 (39.7)	903 (60.3)	1,497 (100)	130 (41)	187 (59)	317 (100)

HR, hormone receptor; HER2, human epidermal growth factor receptor 2; pCR, pathologic complete response; +, positive; −, negative.

### Univariate and Multivariate Analysis of the Correlation Between Clinicopathological Features and Axillary pCR in Patients With the Training Set

Univariate and Multivariate regression predicting nodal pCR *via* training set has been revealed in **Tables 2** and **3**. Univariate analysis revealed that clinical N stage before chemotherapy, the expression level of Ki-67 (OR 2.138, 95% CI 1.694–2.699, P <0.001), and breast pCR status(OR 5.592, 95% CI 4.447–7.031, P <0.001) post-chemotherapy were considerably associated with ypN0 post NCT. The axillary pCR was increased in patients with HR+HER2+ (OR 2.801, 95% CI 2.189–3.585, P < 0.001), HR−HER2+ (OR 4.286, 95% CI 3.2–5.742, P <0.001), HR−HER2− (OR 3.252, 95% CI 2.461–4.297, P <0.001) subtypes compared with HR+HER2− disease, respectively. There was no association between age, menstrual status, site of primary breast tumor, T stage at the time of diagnosis, and axillary pCR (**Table 2**). Multivariable analysis revealed that breast pCR had a strong independent correlation with ypN0 status, with an OR of 4.493 (95% CI 3.409–5.922, P <0.001) for breast pCR *versus* residual breast tumor disease. Relatively more aggressive tumors were also correlated with more chances of ypN0 status, with an OR of 2.409 (95% CI 1.769–3.282, P <0.001) for ER+/HER2+, 3.572 (95% CI 2.501–5.102, P <0.001) for ER−/HER2+, and 2.318 (95% CI 1.610–3.339, P <0.001) for ER−/HER2−, each *versus* ER+/HER2− disease. Lower cN category (OR 0.589 for cN2, OR 0.706 for cN3, each *versus* cN1), elevated expression of Ki-67 (OR = 1.532, 95% CI 1.139–2.061, P = 0.005) *versus* lower expression tumors were correlated with more chances of ypN0 status (**Table 3**).

**Table 2:** Univariate regression predicting nodal pCR in the training set, n (%).

Characteristics		Total	ypN+	ypN0	OR	95% CI	P
Age at diagnosis, years							
≤40		255 (17.8)	145 (56.9)	110 (43.1)			
>40		1,177 (82.2)	724 (61.5)	453 (38.5)	0.866	0.677–1.109	0.254
Menopausal							
Premenopausal		945 (64.2)	561 (59.4)	384 (40.6)			
Postmenopausal		526 (35.8)	332 (63.1)	194 (36.9)	0.869	0.713–1.059	0.165
Quadrant							
Upper outer		639 (51.7)	373 (58.4)	266 (41.6)			0.541
Lower outer		140 (11.3)	90 (64.3)	50 (35.7)	0.917	0.66–1.275	0.607
Lower inner		75 (6.1)	47 (62.7)	28 (37.3)	0.908	0.591–1.396	0.661
Upper inner		269 (21.8)	157 (58.4)	112 (41.6)	1.023	0.789–1.327	0.862
Central		113 (9.1)	75 (66.4)	38 (33.6)	0.725	0.492–1.07	0.105
Clinical T stage							
T1		102 (6.9)	58 (56.9)	44 (43.1)			0.318
T2		866 (58.6)	517 (59.7)	349 (40.3)	0.827	0.577–1.186	0.302
T3		358 (24.2)	220 (61.5)	138 (38.5)	0.745	0.502–1.105	0.143
T4		153 (10.3)	98 (64.1)	55 (35.9)	0.678	0.428–1.073	0.097
Clinical N stage							
N1		745 (50.2)	415 (55.7)	330 (44.3)			<0.001
N2		524 (35.3)	348 (66.4)	176 (33.6)	0.648	0.524–0.802	<0.001
N3		216 (14.5)	133 (61.6)	83 (38.4)	0.799	0.609–1.048	0.105
Ki-67							
Low		373 (27.5)	270 (72.4)	103 (27.6)			
High		983 (72.5)	548 (55.7)	435 (44.3)	2.138	1.694–2.699	<0.001
Subtype							
HR+HER2−		634 (42.8)	477 (75.2)	157 (24.8)			<0.001
HR+HER2+		348 (23.5)	184 (52.9)	164 (47.1)	2.801	2.189–3.585	<0.001
HR−HER2+		240 (16.2)	105 (43.8)	135 (56.3)	4.286	3.2–5.742	<0.001
HR−HER2−		259 (17.5)	127 (49)	132 (51)	3.252	2.461–4.297	<0.001
Breast pCR							
No		1,113 (75.1)	783 (70.4)	330 (29.6)			
Yes		384 (25.9)	120 (31.3)	264 (68.8)	5.592	4.447-7.031	<0.001
Total		1,497	903 (60.3)	594 (39.7)			

HR, hormone receptor; HER2, human epidermal growth factor receptor 2; pCR, pathologic complete response; +, positive; −, negative.

**Table 3:** Multivariate regression predicting nodal pCR in the training set and Point Score.

Characteristics		OR	95% CI	P	Point Score
Clinical N stage					
	N1	1			1
	N2	0.589	0.448–0.773	<0.001	0.5
	N3	0.706	0.495–1.006	0.054	0.5
Ki-67					
	Low	1			1
	High	1.532	1.139–2.061	0.005	1.5
Subtype					
	HR+HER2−	1			1
	HR+HER2+	2.409	1.769–3.282	<0.001	2.5
	HR−HER2+	3.572	2.501–5.102	<0.001	3.5
	HR−HER2−	2.318	1.61–3.339	<0.001	2.5
Breast pCR					
	No				1
	Yes	4.493	3.409–5.922	<0.001	4.5
Total					2–10.5

HR, hormone receptor; HER2, human epidermal growth factor receptor 2; pCR, pathologic complete response; +, positive; −, negative.

### Establish the Scoring System

**Figure 2:**
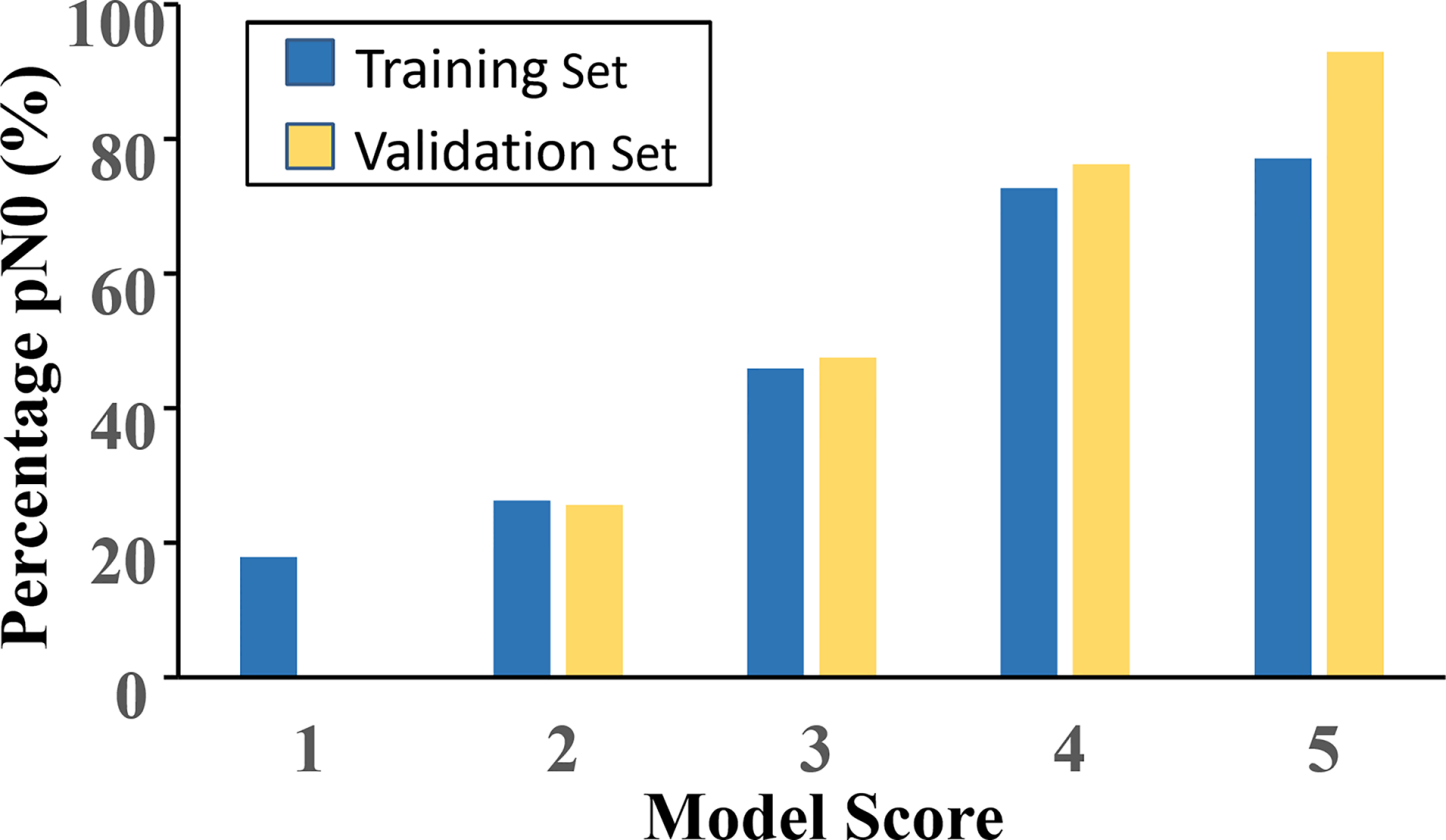
The model score distribution and corresponding ypN0 ratio of the training set and verification set.

According to the value of the OR of each independent predictor, the scoring system has been represented in **Table 3**. In the training set, the total score of all patients was calculated based on the above scoring system. As the score of the cumulative model was ranged from 2 to 10.5, the model adjustment was carried out at a 1–5 numeric scale, as represented in **Table 4**. The model score distribution and corresponding ypN0 ratio of the training set and verification sets are indicated in **Table 4**. Elevated point scores were associated with step by step elevation in the rate of pCR that has been graphically represented in **Figure 2**. The axillary pCR rate of patients with a score of 5 in the training set can reach 77%, while the overall trend of 92.9% in the verification set.

**Table 4:** Basic five-point model score and association with pN0 response in training and validation set, n (%).

OR score	Model score	Training Set, n = 1,497			Validation Set, n = 317		
		ypN0	ypN+	Total	ypN0	ypN+	Total
2–3.5	1	32 (17.8)	148 (82.2)	180 (12)	0	22 (100)	22 (6.9)
4–5.5	2	167 (26.1)	473 (73.9)	640 (42.8)	40 (25.5)	117 (74.5)	157 (49.5)
6–7.5	3	171 (45.8)	202 (54.2)	373 (24.9)	29 (47.5)	32 (52.5)	61 (19.3)
8–9.5	4	167 (72.6)	63 (27.4)	230 (15.4)	48 (76.2)	15 (23.8)	63 (19.9)
10–10.5	5	57 (77)	17 (23)	74 (4.9)	13 (92.9)	1 (7.1)	14 (4.4)

### Effectiveness Evaluation of Scoring System Model

**Figure 3:**
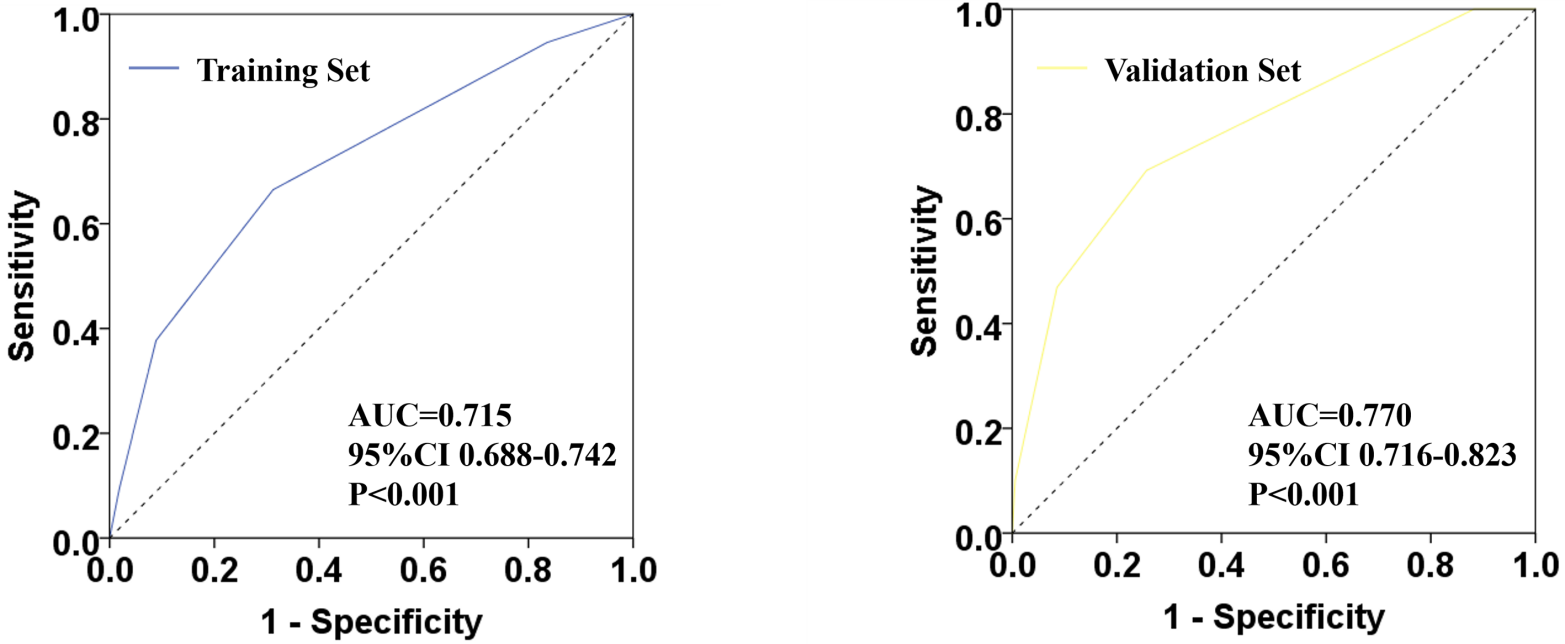
Receiver operating characteristic (ROC) plots for predicting ypN0 status in the training set and verification set.

Based on the model score of all patients, the ROC curve of ypN0 ratio post-NCT was drawn (**Figure 3**). The training set AUC was 0.715 (95% CI 0.688–0.742, P <0.001), and the verification set AUC was 0.770 (95% CI 0.716–0.823, P <0.001) which indicates significant discrimination of the scoring system.

## Discussion

SLNB is the standard method for the staging of the axillary lymph node in patients with early BC and cN0 disease. However, the safety of SLNB is still controversial in patients with initial cN+ stage and downstaging to cN0 after chemotherapy. Earlier studies have been revealed that 22–44% is the rate of axillary pCR post-NCT, which is higher in patients with triple-negative and HER2 positive BC *i.e.*, 40–74% (15, 16). Ideally, doctors will be able to screen out patients who respond well to chemotherapy and have more chances to reach ypN0 post NCT, and suitable to do SLNB after NCT. In this way, the risk of missing positive lymph nodes can be lowered. In the current study, we established a clinically predicted model for patients with cN+ BC for prediction, with good discrimination, pathologically negative nodal status following NCT. The model was established based on the CSCO BC database and independently verified by the BC patient data of Henan Cancer Hospital. The model containing the clinical category of tumor in patients, the expression level of Ki-67, biologic subtype, and breast pCR. Consistent with previous studies, the rate of axillary pCR after NCT is 39.9%, while the training and the verification set is 39.7 and 41%, accordingly. Multivariate analysis revealed that N stage prior to chemotherapy, the expression level of Ki-67, molecular subtype, and breast pCR status after chemotherapy were all independent factors associated with axillary pCR post-chemotherapy. It showed consistency with the results obtained from earlier studies. A 1–5 model scoring system has been constructed on the basis of transformation and summation of the OR values of each variable. To determine the efficiency of the scoring system, the ROC curve of the ypN0 ratio has been plotted post-NCT. The training set AUC is 0.715 and the verification set AUC is 0.770 which indicates the significant discrimination of the scoring system.

In the model scoring system established in this study, breast pCR after chemotherapy accounted for a large weight (4.5 to 10.5). Many earlier studies have been reported that the axillary pCR rate in breast pCR patients has been considerably elevated than that in breast non-pCR patients (17–19). According to Netherlands Cancer Registry, it has been revealed that in newly diagnosed cN+ patients, the axillary pCR rate in breast pCR patients after NCT is 45%, while that in breast non-pCR patients is only 9.4% (20).

Of course, how to judge the state of breast pCR before the operation is one of the problems that need to be solved in the application of this model. Many studies have reported the strategies of predicting breast pCR post NCT, and even the requirement of breast surgery in patients with breast pCR post-NCT has been questioned (21). Image-Guided Minimally Invasive Biopsy(MIB) is expected to precisely predict breast pCR. A single-center prospective study from MD Anderson Cancer Center involved 40 patients with clinical T1-3N0-3M0 and TN or HER2-positive BC who were assessed as complete or partial remission by ultrasound or mammography after NCT and underwent fine-needle aspiration biopsy or coarse needle biopsy before the operation. The accuracy for prediction breast pCR can reach 98.0% with a lower FNR of 5.0% by a combination of the two invasive biopsy methods (22). Another prospective, a monocenter cohort study has also shown that a vacuum-assisted MIB can accurately diagnose a pCR provided that the pathological evaluation shows a representative sample (23). At present, although pathologic response in the breast is not available preoperatively during the time of surgical decision-making. However, these studies suggested that MIB has a good prospect in predicting the clinical application of breast pCR (22, 23).

Numerous studies have been established models for the prediction of ypN0 post-NCT, but almost all of them are single-center or multi-center small sample data, and only a few of the models are based on NCDB large database. The most recent model reported in 2018 was constructed with 19,115 (70% being assigned as “testing cohort” to created initial model and 30% being assigned as “validation cohort” to confirmation of model strength) clinically node-positive BC patients who underwent NCT and then received breast surgery and dissection of the axillary lymph node. The model was carried out to predict pathologically node-negative status. The study revealed that age, histological type, initial N stage, histological grade, molecular classification, and breast pCR status were independently predicted ypN0 post NCT. The AUC of the training set and verification set were 0.781 and 0.788, accordingly (12). Moreover, data of the training, as well as verification set, have been obtained from the NCDB database, which lacks external independent data verification. While Murphy et al. also validated the model independently by external data of Mayo Clinic, the sample size in the validation set seems to be not enough (n = 180) (13). The advantage of our model is that the model was established based on China’s authoritative CSCO BC database (training set), and independently verified by the BC patient data of Henan Cancer Hospital (validation set). Although tumor histology and grade are not included in our model, we get similar AUC values. The training set AUC is 0.715, the verification set AUC is 0.770 which reveals that the prediction model on the basis of the underlined scoring system is stable with good discrimination. This may also suggest that our model may be more convenient for the Asian population.

If we can use primary data, more parameters were able to be included in our model, such as tumor histology and grade, MRI or ultrasound or clinical tumor response, and chemotherapy details. Thus, our model may be greatly improved. The information is incomplete or not available in the CSCO BC database, and therefore, was not included in the model. However, our model was based on a larger sample of the Asian population database, also the largest sample size, for the first time. We, therefore, included a more representative and heterogenous cohort of patients from across China and hospitals of varying sizes and varying practice settings.

The model of the current study may provide a reliable screening method for patients who are suitable to do SLNB after NCT with initial cN+ disease. For patients that were at risk of node-negative, axillary staging with SLNB surgery would be recommended, while for patients that were at higher risk of nodal positive disease for a longer period, ALND may be considered. For example, the chances of ypN0 for patients with a score of 1 point is less than 20%, and it should be careful to do SLNB, or even direct dissection of the axillary lymph node is recommended. For patients with a score of 2–3 points, the probability of ypN0 is 20–50%. SLNB can be considered. The chances of ypN0 for patients with a score of 4–5 points is more than 70%, and direct SLNB is recommended. Hence, the underlined model permits surgeons for SLNB surgery in patients with more chances of nodal response to NCT, so reducing the chances of false-negative events.

The model established in this study is based on the authoritative BC registration database in China and verified by the independent data of Henan Cancer Hospital. As far as we know, this is the first clinically predicted model for ypN0 on the basis of the Asian population database. The shortcomings of this study included that it is a retrospective study that is based on the database. There is a large number of missing data, including chemotherapy regimens. So, data of many patients were excluded from the study. Notably, chemotherapy regimens are not included in both of our model and models based on NCDB (12, 13). A lower axillary pCR rate was reported in patients treated with a taxane without an anthracycline (23.7%) than an anthracycline without a taxane (19%) (15). Unfortunately, we cannot rule out the impact of chemotherapy regimens. But it also reflects that this study is more in line with the characteristics of real-world patients. We also suggested that a large heterogeneous cohort of patients used for generating models makes them universally appropriate for patients at all medical centers. A multicenter BC NCT database containing much more variables is being established, and we hope to improve the model in the future.

In brief, we established and confirmed a model to predict ypN0 post-chemotherapy in newly diagnosed cN+ patients which has good accuracy and efficacy. The models, which included patient clinical nodal category, Ki-67 expression, biologic subtype, and breast pCR, showed good discrimination. This clinically useful model is helpful to the reasonable choice of axillary surgery after NCT and reduces the risk of missing positive lymph nodes in SLNB after NCT.

## Data Availability Statement

The datasets presented in this article are not readily available because at present, the CSCO BC database is not a fully public database. Requests to access the datasets should be directed to zlyyliuzhenzhen0800@zzu.edu.cn.

## Ethics Statement

The authors are responsible for the current study in confirming that queries associated with the precision or reliability of the underlined work are properly evaluated and solved. The current study was performed according to the Declaration of Helsinki (as revised in 2013). The approval for the underlined study was provided by the Ethical Review Committee of the Affiliated Cancer Hospital of Zhengzhou University (No. 2019001). As the current study was retrospectively planned, informed consent was waived *via* Affiliated Cancer Hospital of Zhengzhou University.

## Author Contributions

JjZ performed the data analyses and wrote the manuscript. ZzL contributed to study design and editing of the manuscript. All authors helped in the data collection. All authors contributed to the article and approved the submitted version.

## Funding

The underlined study has been supported *via* the Science and Technology Development Plan of Henan Province (202102310415).

## Conflict of Interest

The authors declare that the research was conducted in the absence of any commercial or financial relationships that could be construed as a potential conflict of interest.
